# Hungry pigeons prefer sooner rare food over later likely food or faster information

**DOI:** 10.3389/fpsyg.2024.1426434

**Published:** 2024-06-24

**Authors:** Neslihan Wittek, Berna Selin Sayin, Nurdem Okur, Kevin Wittek, Naciye Gül, Fatma Oeksuez, Onur Güntürkün, Patrick Anselme

**Affiliations:** Biopsychology department, Ruhr University Bochum, Bochum, Germany

**Keywords:** motivation, information, effort, preference, pigeon, reinforcement schedule

## Abstract

**Introduction:**

Making decisions and investing effort to obtain rewards may depend on various factors, such as the delay to reward, the probability of its occurrence, and the information that can be collected about it. As predicted by various theories, pigeons and other animals indeed mind these factors when deciding.

**Methods:**

We now implemented a task in which pigeons were allowed to choose among three options and to peck at the chosen key to improve the conditions of reward delivery. Pecking more at a first color reduced the 12-s delay before food was delivered with a 33.3% chance, pecking more at a second color increased the initial 33.3% chance of food delivery but did not reduce the 12-s delay, and pecking more at a third color reduced the delay before information was provided whether the trial will be rewarded with a 33.3% chance after 12 s.

**Results:**

Pigeons’ preference (delay vs. probability, delay vs. information, and probability vs. information), as well as their pecking effort for the chosen option, were analyzed. Our results indicate that hungry pigeons preferred to peck for delay reduction but did not work more for that option than for probability increase, which was the most profitable alternative and did not induce more pecking effort. In this task, information was the least preferred and induced the lowest level of effort. Refed pigeons showed no preference for any option but did not drastically reduce the average amounts of effort invested.

**Discussion:**

These results are discussed in the context of species-specific ecological conditions that could constrain current foraging theories.

## 1 Introduction

To survive in their environment, animals should decide for the motivationally most salient cues, approach them, and invest effort to obtain the expected resources and maximize reward procurement (Herrnstein, 1961; Charnov, 1976). Thus, if odor A is associated with a highly appetitive reward and odor B with a poorly appetitive reward, animals should follow odor A, compared to B. They should also prefer odor A to B. These are the basic predictions of incentive salience theory, which posits that a cue predictive of reward may become motivationally attractive compared to a reward-unrelated cue—in addition to the ability to learn the cue-reward association (Berridge, 2007). As a result, cues imbued with incentive salience generate more responses and are preferred to unattractive cues. Indeed, if a reward cue induces more or faster responses than another one, it is usually preferred in free-choice tasks (Shapiro et al., 2008; but see Williams, 1992). Discounting experiments also indicate that an immediate and certain reward is more preferred and generates more responses than a reward obtained after a longer delay or with a lower probability (Mazur, 2000; Green et al., 2014; Hayden, 2016).

Although incentive salience theory predicts consistency between performance and cue preference, unexpected relationships between cue attraction, effort, and choice have been reported—especially under reward uncertainty. In foraging experiments, for example, animals may spend more (or, at least, a significant portion of) time and effort trying to obtain unguaranteed food although the same food is available galore (Forkman, 1996; Andrews et al., 2015). Also, humans pay for information about reward delivery, even when the information is irrelevant to the outcome (Bennett et al., 2016; Rodriguez Cabrero et al., 2019; Liew et al., 2023). Similarly, pigeons and starlings accept to sacrifice food for information in a task, even if this information does not change the outcome (Spetch et al., 1990; Stagner and Zentall, 2010; Vasconcelos et al., 2015). Given that preference, effort, and reward rate do not always work in an easily predictable way (e.g., Anselme et al., 2022, 2024), we confronted pigeons with the option to choose a shorter delay to food, a higher probability of food, or a sooner information about the trial outcome under food restriction or refeeding conditions. How would they decide? Incentive salience theory does not make clear predictions. Obtaining food sooner or with a higher probability should be attractive, but revealing a cue announcing how the trial will end might also be a source of motivation.

In addition to comparing several reinforcement schedules in a within-subject design, our experiment aimed to test the limits of how information can motivate choices and responses in pigeons, depending on their food restriction level and the work required to obtain information. First, there is evidence that animals allowed to choose between immediate food and the same food following moderate effort tend to favor the latter option, a preference amplified under a low food restriction level (Inglis et al., 1997). But these tasks—and others in which a preference for an informative option typically emerges (e.g., Zentall, 2016)—often require limited investment to get the trial outcome. In the wild, however, animals should invest much time and effort looking for useful signals (information) in the vicinity, especially if they are hungry—e.g., during the winter period. Second, an informative stimulus is not just a stimulus that precedes reward procurement, it is a stimulus that disambiguates a context in which reward is not guaranteed. And this stimulus is more likely to become an information and be preferred if there is a longer time gap between its occurrence and the outcome (e.g., Cunningham and Shahan, 2019; Fraser and Holland, 2019). How would pigeons under a low or high food restriction level work to obtain sooner information through pecking? Incentive salience theory predicts that they should work harder for information while hungry, but the main question here is to determine whether they would work harder for information than for food while not hungry.

We exposed pigeons (*Columba livia*) to three distinct predictive cues with only two cues being simultaneously displayed during choice trials. If a pigeon did not peck at a presented cue, the three options were equivalent: The trial ended after a fixed delay (12 s) and provided food with a relatively low probability (0.33). This was the Pavlovian component of the task, as no action was required to obtain a food reward on successful trials. Depending on the cue, pecking activity had the effect of reducing the duration of the delay to food, or increasing the probability of food, or obtaining sooner disambiguation of the trial outcome. These effects were directly proportional to the number of pecks. The first two represented the instrumental component of the task, since pecking could shorten the delay or increase reward probability. In contrast, pecking to obtain information might have been non-instrumental since it satisfied curiosity without changing anything about reward delivery—but we cannot exclude that the conditioned reinforcing properties of the reward induced instrumental responding as well (Parkinson et al., 2005). The originality of this task was to ask pigeons to select their preferred values of delay reduction, probability increase, and information gathering.

## 2 Materials and methods

### 2.1 Animals and housing conditions

Twelve adult homing pigeons (9 females) were obtained from local breeders and had already been involved in an unrelated experiment. They were maintained at 85–90% of their free-feeding body weight (480 ± 8.2 g). Water was accessible *ad libitum* and additional food supply was provided in their home cage, at least 30 min after each daily session and before the weekends. The pigeons were individually housed under a 12 h light/dark cycle (lights on at 7:30 a.m.). All procedures were approved by the ethics commission of the State of North Rhine-Westphalia (81-02.04.2023.A006), Germany, and followed the European Communities Council Directive 86/609/EEC concerning the care and use of animals for experimentation.

### 2.2 Apparatus

Pigeons were tested in individual operant chambers (34 cm width × 34 cm depth × 32 cm height), equipped with a white house light. In the middle of the front panel, two transparent pecking keys (4 cm × 4 cm), coupled with an electric switch, allowed the animal to respond to a stimulus displayed on an LCD flat screen located behind the panel. A rotating feeder located below the two keys, provided access to food pellets after the presentation of a rewarded stimulus. The house light remained on during the intertrial interval (ITI) and the food port light was on during each food delivery. Pigeon activity could be monitored with a camera fixed on the back panel of the Skinner box. A custom-written Matlab R2019b code using the Biopsychology Toolbox controlled the apparatus (Rose et al., 2008).

### 2.3 Procedure

#### 2.3.1 Pretraining

The pigeons were exposed to five stimuli (orange, purple, green, white, gray), presented randomly and one per trial on either response key (left or right). Each orange, purple, and green stimuli were presented for 12 s and terminated with a 0.8 probability of food delivery (2 pellets). Each white and gray stimuli were also presented for 12 s, but the white stimulus was followed by food delivery with a probability of 1 (2 pellets) and the gray stimulus was never followed by food. Food delivery was independent of the animal’s responses to the stimuli. Each stimulus was shown 6 times per response key within a daily session, for a total of 60 presentations (trials) per session. A session always started and ended with a 20-s ITI (range: 10–30 s). Pretraining stopped as soon as the pigeons pecked at each stimulus, to avoid habit formation in this first phase.

#### 2.3.2 Training

**Figure 1:**
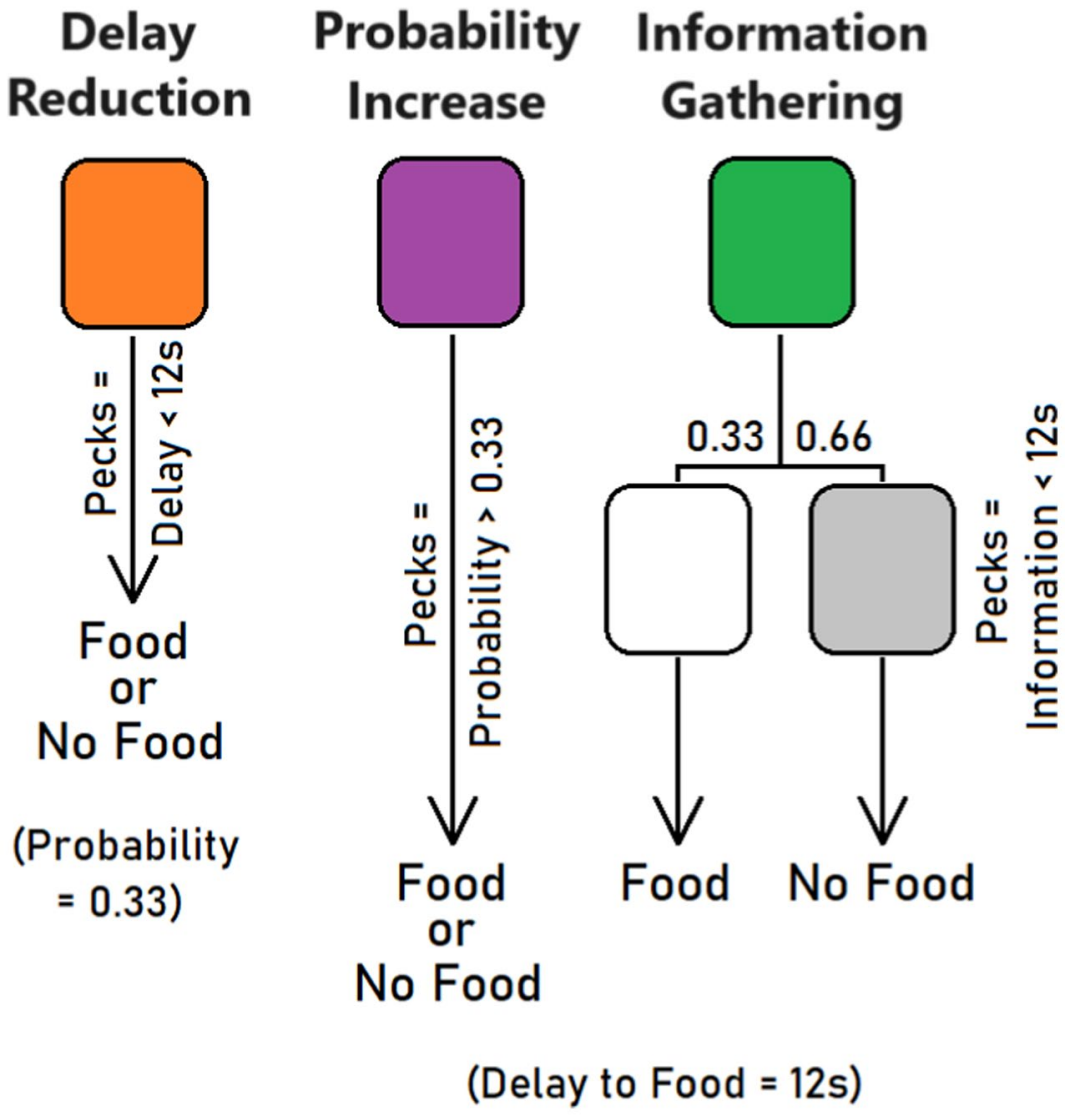
Conditions used in the training and test phases. The pigeons had the opportunity to peck at an illuminated key to reduce delay to food (delay reduction), increase probability of food (probability increase), or shorten the waiting time until a white or gray stimulus made it clear whether food will be provided or not.

Only one stimulus per trial was presented (forced trials), and it could randomly occur on the left or right key. In this new phase, orange, purple, and green stimuli had distinct effects (counterbalanced across pigeons). One stimulus, say orange, signaled the option for delay reduction (Figure 1). It was presented for 12 s and, in the absence of pecks, two food pellets were delivered with a 0.33 probability. However, the pigeon could reduce the 12-s delay by 0.5 s per peck at the orange illuminated key. For example, a pigeon that pecked 10 times at the key obtained a decrease in stimulus presentation time from 12 s to 12 − (10 × 0.5) = 7 s. The probability of food after a reduced delay remained 0.33. Another stimulus, say purple, was associated with reward probability increases. Again, the stimulus was presented for 12 s and delivered two food pellets with a 0.33 probability in the absence of pecks. But the pigeon could increase food probability by 0.015 per peck at the purple illuminated key. For example, a pigeon that pecked 26 times at the key could increase the probability of food delivery from 0.33 to 0.33 + (26 × 0.015) = 0.72. The delay to reward remained 12 s. Finally, a third stimulus, say green, was equivalent to the other two at start (12-s delay, 0.33 probability of two food pellets) but offered the option to be informed earlier about the outcome. Each peck reduced the time by 0.5 s before a stimulus (white or gray) appeared and signaled if reward would come or not. While the white stimulus signaled that the trial will be rewarded, the gray stimulus predicted the absence of reward. For example, a pigeon that pecked 10 times at the green stimulus had its presentation time decreased from 12 s to 12 − (10 × 0.5) = 7 s. Then, the bird saw a second white or gray stimulus for 5 s before food was delivered or not, respectively. Of note, in the absence of pecks, the white or gray stimulus was shown for less than a second, just before food was delivered or not. Contrary to the other two conditions, the delay to food remained of 12 s and food probability of 0.33. Overall, 8 orange, green, and purple stimuli were shown on each response key on a random basis within a session, for a total of 48 forced trials. A variable ITI of 30 s was used (range: 15–45 s).

#### 2.3.3 Test

In addition to the 48 forced trials, the pigeons were exposed to 24 choice trials, randomly distributed within each daily session. In these trials, the pigeons could freely choose between delay reduction and probability increase, or delay reduction and information gathering, or probability increase and information gathering (8 choices of each type per session, counterbalanced for key location). Choosing one option turned the other key off and the trial continued as if it was a forced trial (see training phase). Our analyses rest on these choice trials, the forced trials being used to constantly refresh memory about the available options throughout a session. Specifically, we were interested in the preference of pigeons for the different options depending on their food restriction status. Thus, the test phase was conducted in a pre-treatment block under food restriction for 12 sessions to determine a baseline, a treatment block after refeeding for 6 sessions, and a post-treatment block under food restriction again for 6 sessions. There were approximately 2 weeks between the three blocks, a time necessary to allow the pigeons to gain or lose the appropriate weight.

### 2.4 Statistics

As we used a within-subject design, the statistical analyses were carried out by means of two-tailed repeated measures ANOVAs (Statistica 14). We compared the total number of choices between the three options, all sessions combined. We also analyzed the number of choices per session for the two options in competition. Finally, we compared pecking activity for one option relative to the other two options. Statistical significance was accepted at *p* < 0.05. Effects sizes were reported. Means and standard errors were used for all calculations.

## 3 Results

### 3.1 Total number of choices

**Figure 2:**
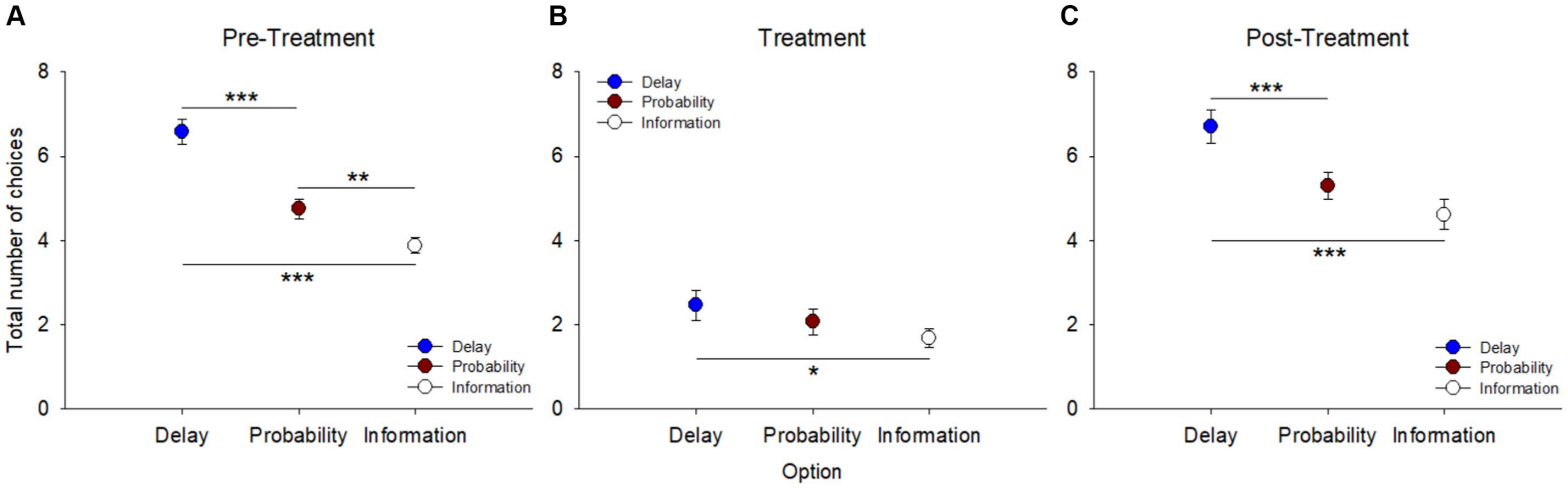
Total number of choices for each option (delay reduction, probability increase, and information gathering), irrespective of the alternative option, across sessions in the three blocks: **(A)** pre-treatment, **(B)** treatment, and **(C)** post-treatment. Choices between options were not independent of each other. **p < 0.01, ***p < 0.001.

During pre-treatment (Figure 2A), the total number of choices for one alternative across the 12 sessions and irrespective of the other alternatives, showed a preference for delay reduction over probability increase [*F*(1, 143) = 40.761, *p* < 0.001, η^2^_p_ = 0.22] and information gathering [*F*(1, 143) = 82.559, *p* < 0.001, η^2^_p_ = 0.37]. There was also a preference for probability increase compared to information gathering, despite a low effect size [*F*(1, 143) = 11.127, *p* = 0.001, η^2^_p_ = 0.07].

After the pigeons were refed to regain their baseline body weight, they were tested using the same procedure for 6 sessions. Over this treatment period, they almost lost their motivation to respond to the task (Figure 2B). Also, only the preference for delay reduction over information gathering remained significant [*F*(1, 71) = 5.808, *p* = 0.018, η^2^_p_ = 0.07]. Following a new food restriction period for post-treatment (Figure 2C), the pigeons retrieved their initial preference for delay reduction over probability increase [*F*(1, 71) = 14.852, *p* < 0.001, η^2^_p_ = 0.17] and continued to prefer delay reduction to information gathering [*F*(1, 71) = 20.902, *p* < 0.001, η^2^_p_ = 0.23]. However, no significant difference was found between probability increase and information gathering [*F*(1, 71) = 2.722, *p* = 0.103, η^2^_p_ = 0.04].

### 3.2 Delay reduction vs. probability increase

**Figure 3:**
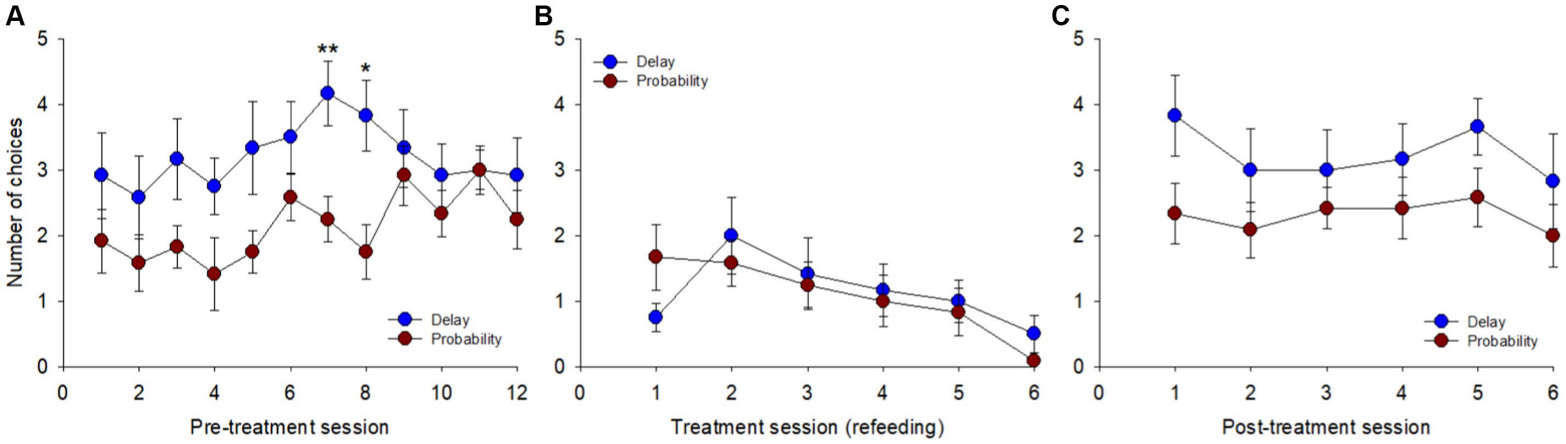
Number of choices between the cues for delay reduction and probability increase across sessions in the three blocks: **(A)** pre-treatment, **(B)** treatment, and **(C)** post-treatment. *p < 0.05, **p < 0.01.

A session-per-session analysis of the number of choices between delay reduction and probability increase (presented simultaneously on distinct keys) was carried out. During pre-treatment (Figure 3A), there was an overall significant effect of day on the number of choices [*F*(23, 253) = 2.802, *p* < 0.001, η^2^_p_ = 0.20], both found for delay reduction [*F*(1, 11) = 62.743, *p* < 0.001] and probability increase [*F*(1, 11) = 65.011, *p* < 0.001], but without any clear trends across days. The pigeons showed an overall preference for delay reduction relative to probability increase [*F*(1, 11) = 5.061, *p* = 0.046], with significant differences on days 7 (*p* = 0.009) and 8 (*p* = 0.018) and non-significant trends on days 3, 4, and 5 (*p*s = 0.058).

Treatment reduced the number of choices made (Figure 3B), an effect more and more pronounced across the 6 sessions [*F*(11, 121) = 3.093, *p* = 0.001, η^2^_p_ = 0.22], both for delay reduction [*F*(1, 11) = 14.412, *p* = 0.003] and probability increase [*F*(1, 11) = 13.903, *p* = 0.003]. The overall comparison between the two options was non-significant [*F*(1, 11) = 0.072, *p* = 0.793]. At post-treatment (Figure 3C), the pigeons chose more often again and performance was stable across the 6 sessions [*F*(11, 121) = 1.294, *p* = 0.236, η^2^_p_ = 0.10]. Pigeons chose delay reduction more often than probability increase each day, on average, and only the overall comparison between the two options was significant [*F*(1, 11) = 6.742, *p* = 0.025].

### 3.3 Delay reduction vs. information gathering

**Figure 4:**
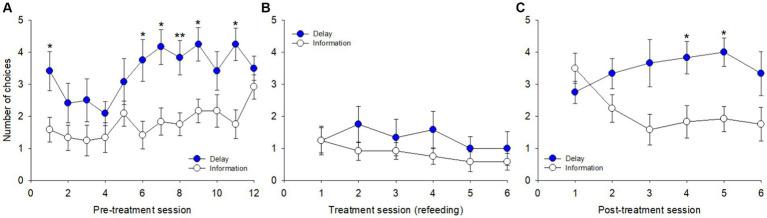
Number of choices between the cues for delay reduction and information gathering across sessions in the three blocks: **(A)** pre-treatment, **(B)** treatment, and **(C)** post-treatment. *p < 0.05, **p < 0.01.

In the pre-treatment block (Figure 4A), a significant overall effect of day on the number of choices was shown [*F*(23, 253) = 4.844, *p* < 0.001, η^2^_p_ = 0.30], a significant increase in preference being observed both for delay reduction [*F*(1, 11) = 71.699, *p* < 0.001] and probability increase [*F*(1, 11) = 49.553, *p* < 0.001] across days. The pigeons preferred delay reduction over information gathering [*F*(1, 11) = 9.690, *p* = 0.010], with significant differences on days 1 (*p* = 0.025), 6 (*p* = 0.025), 7 (*p* = 0.010), 8 (*p* = 0.006), 9 (*p* = 0.027), and 11 (*p* = 0.010).

During treatment (Figure 4B), the number of choices slightly decreased but the overall effect of day was not significant [*F*(11, 121) = 1.076, *p* = 0.386, η^2^_p_ = 0.09]. The comparison between the two options was also not significant [*F*(1, 11) = 1.644, *p* = 0.226]. In the post-treatment block (Figure 4C), a significant overall effect of day on the number of choices was shown [*F*(11, 121) = 3.062, *p* = 0.001, η^2^_p_ = 0.22], which consisted of an increase in preference across sessions for delay reduction [*F*(1, 11) = 185.744, *p* < 0.001] and of a decrease in preference for information gathering [*F*(1, 11) = 51.072, *p* < 0.001]. The overall comparison between the two options was significant [*F*(1, 11) = 7.792, *p* = 0.017], and pairwise comparisons were significant on days 4 (*p* = 0.019) and 5 (*p* = 0.012) and barely non-significant on day 3 (*p* = 0.052).

### 3.4 Probability increase vs. information gathering

**Figure 5:**
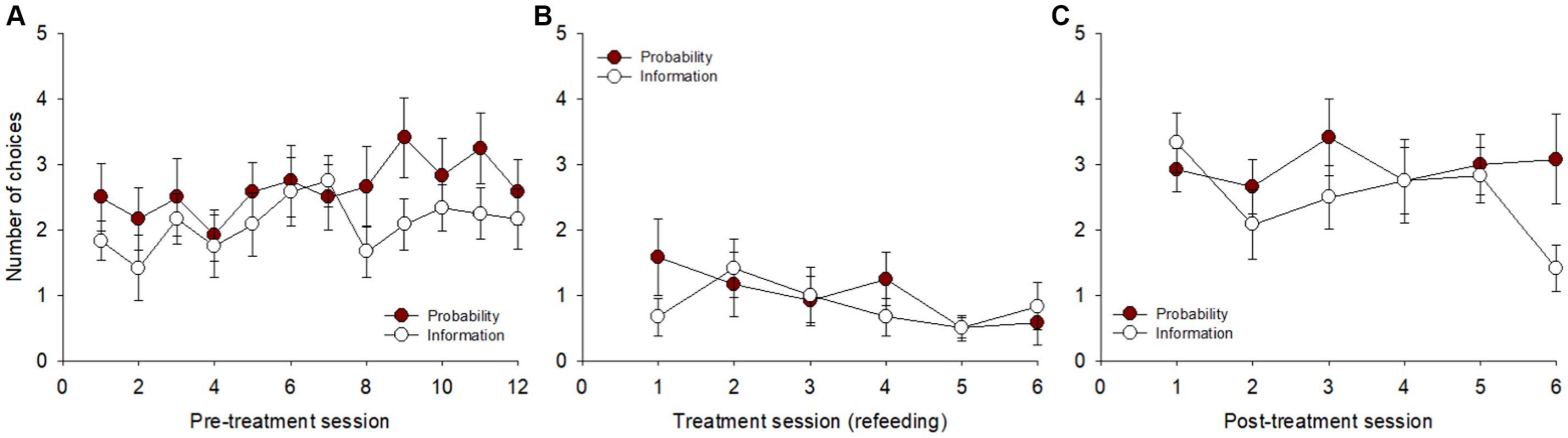
Number of choices between the cues for probability increase and information gathering across sessions in the three blocks: **(A)** pre-treatment, **(B)** treatment, and **(C)** post-treatment.

A comparison of probability increase and information gathering during pre-treatment (Figure 5A) showed no overall significant effect of day on the number of choices [*F*(23, 253) = 1.397, *p* = 0.111, η^2^_p_ = 0.11] and no overall preference between the two options [*F*(1, 11) = 2.560, *p* = 0.138].

In the treatment block (Figure 5B), the overall effect of day was also not significant [*F*(11, 121) = 1.233, *p* = 0.273, η^2^_p_ = 0.10], as well as the preference between the two options [*F*(1, 11) = 0.301, *p* = 0.594]. Similarly, in the post-treatment block (Figure 5C), there were non-significant overall effects of day [*F*(11, 121) = 1.265, *p* = 0.252, η^2^_p_ = 0.10] and of preference between the two options [*F*(1, 11) = 1.058, *p* = 0.326].

### 3.5 Selected values per option

In addition to counting the number of choices, we also wanted to report the actual mean values of reduced delay, increased reward probability, and faster information, selected by the pigeons during choice trials and across sessions. Table 1 shows, for each block, the selected values for each reinforcement condition relative to the alternative reinforcement conditions. As reported, hungry pigeons approximately reduced the 12-s delay by a half through pecking with the delay reduction option but were less prone to work with respect to information gathering. For example, during pre-treatment, they reduced the stimulus duration to 6.70 s or 6.53 s (depending on the alternative option). But they were exposed to the information stimulus for only 3.88 s or 3.97 s (depending on the alternative option), suggesting limited work upfront to obtain disambiguation. They also approximately doubled the initial 0.33 probability of food through pecking with the probability-increase option. Those effects appeared slightly attenuated during treatment, with refed pigeons. As also mentioned in Table 1, pecking activity for a selected option was statistically equivalent whatever the alternative.

**Table 1:** Selected values for each selected option relative to the alternative reinforcement options in the pre-treatment, treatment, and post-treatment blocks.

Block	Selected option	Alternative	Selected value (MSE)	*p*-value	Effect size (η^2^_p_)
Pre-treatment	Delay	Probability Information	6.70 s (0.18) 6.53 s (0.18)	0.678	<0.01
	Probability	Delay Information	0.65 (0.018) 0.66 (0.016)	0.618	<0.01
	Information	Delay Probability	3.88 s (0.23) 3.97 s (0.20)	0.805	<0.01
Treatment	Delay	Probability Information	7.09 s (0.33) 6.99 s (0.28)	0.987	<0.01
	Probability	Delay Information	0.55 (0.028) 0.59 (0.024)	0.495	0.01
	Information	Delay Probability	2.72 s (0.35) 3.34 s (0.42)	0.348	0.03
Post-treatment	Delay	Probability Information	5.80s (0.27) 6.03 s (0.23)	0.569	<0.01
	Probability	Delay Information	0.76 (0.032) 0.70 (0.021)	0.117	0.04
	Information	Delay Probability	4.99 s (0.33) 5.01 s (0.26)	0.986	<0.01

The *p*-values refer to a comparison of the selected values for the selected option between the other two alternatives.

To reach the pre-treatment selected values, the pigeons gave approximately 10–11 pecks at the delay-reduction cue, 21–22 pecks at the probability-increase cue, and 7–8 pecks at the information-gathering cue. Although the pigeons chose delay reduction more often, they seemed to put more effort in increasing the probability of food. However, such a conclusion is incorrect. They could peck at a key for 12 s to increase probability but the time available was shorter to reduce a delay, because some pecking time was used to do it—whether to obtain sooner food or sooner information. Based on the average time values and their corresponding number of pecks reported above, we can estimate the number of pecks per second for each option. So, the pigeons gave ((10 + 11)/2)/(6.70 + 6.53)/2 = 1.59 pecks per second for delay reduction, ((21 + 22)/2)/12 = 1.79 pecks per second for probability increase, and ((7 + 8)/2)/(12 − (3.88 + 3.97)/2) = 0.93 pecks per second for information gathering. These values were very similar and their differences represented much less than a peck per second, especially between the delay-reduction and probability-increase options.

In the treatment block, the pigeons gave ((9 + 10)/2)/(7.09 + 6.99)/2 = 1.35 pecks per second for delay reduction, ((15 + 17)/2)/12 = 1.33 pecks per second for probability increase, and ((5 + 7)/2)/(12 − (2.72 + 3.34)/2) = 0.67 pecks per second for information gathering. Finally, in the post-treatment block, the estimated number of pecks per second for each option was ((12 + 13)/2)/(5.80 + 6.03)/2 = 2.11 for delay reduction, ((25 + 29)/2)/12 = 2.25 for probability increase, and ((9 + 10)/2)/(12 − (4.99 + 5.01)/2) = 1.36 for information gathering.

### 3.6 Profitability per peck

To determine the optimality of investing quite similar effort in each selected option, we calculated the average ratios of the chance of food to unit time per trial for the two keys in competition, which represent an estimate of peck-induced profitability. During pre-treatment, for delay reduction, the measured average ratio was (0.33/6.70 + 0.33/6.53)/2 = 0.049% per second. In other words, if reducing the delay was vital for the animal (e.g., because of competition for resources), in the present testing conditions and with the observed pecking rate, the chance of eating would increase by 0.049% every passing second. The peck-induced profitability ratio for information gathering was lower: (0.33/(12–3.88) + 0.33/(12–3.97))/2 = 0.041% every passing second. In contrast, the ratio calculated for probability increase was higher: (0.65/12 + 0.66/12)/2 = 0.055% every passing second. These results indicate that increasing food probability through pecking effort was the optimal strategy. But this was not what the pigeons did since our results reveal that no more effort was invested for probability increase than for delay reduction and that probability increase was not preferred to delay reduction.

The same calculations in the treatment block indicated that peck-induced profitability for delay reduction was (0.33/7.09 + 0.33/6.99)/2 = 0.046% every passing second. Profitability for information gathering was (0.33/(12–2.72) + 0.33/(12–3.34))/2 = 0.036% every passing second. Finally, profitability for probability increase was: (0.55/12 + 0.59/12)/2 = 0.047% every passing second. The optimal strategy was to preferentially choose and work for probability increase in the task, although the difference with delay reduction was less pronounced. We saw no more effort invested and no more choices for probability increase than for delay reduction. Of course, the pigeons were not hungry in this block but yet they pecked at the response keys, with selected values of pecks per second only slightly reduced compared to pre-treatment.

In the post-treatment block, peck-induced profitability was (0.33/5.80 + 0.33/6.03)/2 = 0.056% per passing second for delay reduction. It was (0.33/(12–4.99) + 0.33/(12–5.01))/2 = 0.047% every passing second for information gathering. Finally, it was (0.76/12 + 0.70/12)/2 = 0.060% every passing second for probability increase. These values were all higher than during pre-treatment, suggesting complete reversal of the food restriction-induced behavioral effects. Again, increasing food probability through pecking effort was the optimal strategy, although the pigeons did not invest much more effort in this option and chose it less often than delay reduction.

## 4 Discussion

In summary, we found that hungry pigeons (pre- and post-treatment blocks) preferred the delay-reduction option over the probability-increase and the information-gathering options, and they were indifferent between these latter two options. With respect to the pecking effort made once an option was selected, pigeons worked comparably to reduce delay or increase probability, and they tended to work a bit less for sooner information. Given that food expectation was higher in the probability-increase option, preference and pecking effort were clearly not a function of food expectation—i.e., not optimal. Refeeding the pigeons (treatment block) created indifference between the three options and only slightly reduced pecking effort in these options, but pecking effort remained insensitive to food expectation. The reversal of the behavioral effects found after reintroducing food restriction in the post-treatment block showed that food restriction influenced preference and effort. Of note, this experiment was conducted mostly with female pigeons, so potential sex differences might exist in the preferences and effort measured.

Given the long delay to food (12 s) and/or the relatively low food probability (0.33), temporal and probabilistic discounting should play a role in accounting for our results (McKerchar and Renda, 2012; Green et al., 2014). From a discounting perspective, paying less attention to the information-gathering option (both in terms of effort and choice) was rational because no action could improve the conditions of reward delivery. Even though there is a willingness of human and nonhuman animals to work for non-instrumental information in other tasks, collecting this information was often tested by giving the individuals the option to immediately receive information by a single response (e.g., Zentall, 2016; Dunn et al., 2024). In our experiment, however, the pigeons had to work harder for sooner information. Accordingly, the information stimulus was delayed, and delaying information for a few seconds is sufficient to reduce its attractiveness (McDevitt et al., 1997). The relatively low probability of food should not have been a source of demotivation for that option because, in effortless tasks, information continues to be preferred to food by pigeons and starlings despite probabilities much lower than 0.33 (e.g., Vasconcelos et al., 2015; Fortes et al., 2016).

Our informative option differed radically from the informative option in most experimental procedures, such as the suboptimal choice task. In this task, an informative stimulus is shown following a single peck and is more strongly preferred (to a non-informative option) when the waiting time before food delivery is longer (e.g., Spetch et al., 1990; Cunningham and Shahan, 2019; Alba et al., 2021). Indeed, the advance notice of the reward is only meaningful if there is a time gap between information and reward delivery. Here, our goal was not to reproduce the traditional information-seeking paradigms, where the conditions of stimuli presentations are fixed in advance. We aimed to determine the extent to which pigeons would peck to shorten the delay for information and hence make information meaningful. This effect was mostly expected in the treatment block (refeeding), because refed pigeons might be less focused on food delivery. However, in our task, pecking rates for information remained low in all blocks, so information did not occur soon and therefore was not really useful.

What about delay reduction and probability increase from a discounting approach? The results are perplexing because we are faced with a paradox between effort and preference. Our pigeons pecked similarly for delay reduction and probability increase but they preferred the former to the latter option. The preference for a reduced delay is predicted by the discounting paradigm, since an immediate reward has a higher incentive/subjective value than a delayed reward (McKerchar and Renda, 2012; Green et al., 2014). A likely reward should obviously also be more attractive than a less likely one. But when minding the ecology of pigeons, a reduced delay weighs more strongly than an increased probability: Pigeons live in flocks and constantly look out for food. So, their main concern should be to find food *before* potential competitors do. This ecological constraint functionally explains why delays are so aversive for pigeons with their competitors always in physical proximity (e.g., Mazur and Biondi, 2009). Nevertheless, it is worth mentioning that delays are also aversive to other species. Gabriel et al. (2021) showed that rats prefer obtaining one food pellet for sure following one lever press over obtaining one food pellet with a 0.25 probability following one lever press. But when the delay to food was increased over a session (from 0 to 7.5 s), preference shifted toward the risky option. These findings are consistent with the evidence that delay reduction may be preferred to a high probability of food.

This reasoning could clarify the preference for delay reduction over reward probability increase. However, contrary to the predictions of incentive salience theory, our pigeons invested comparable effort in both situations. In fact, the average of pecks per second was even slightly lower for delay reduction (1.59) than for probability increase (1.79). If delay reduction is the optimal foraging strategy for pigeons in the wild, why effort was similar in the two options is unclear. In this task, they could also have favored the most profitable one—i.e., reward probability increase (0.055% peck-induced profitability per second) compared to delay reduction (0.049%)—but then a preference for it would have been expected. Despite the opportunity to increase food probability significantly, this strategy did not motivate choice and effort more than delay reduction. A reasonable explanation for this fact is that whatever the option chosen, some irreducible uncertainty remained. In the probability-increase option, reaching a food probability of 1 required considerable effort that was difficult to estimate and the aversive long delay could not be shortened. In the delay-reduction option, there was residual delay induced by pecking requirements and the low probability of food could not be altered. Irreducible uncertainty may have led the pigeons to respond similarly in both options. However, this suggestion remains speculative without varying the delay decrement values and the probability increment values per peck to study the dynamics of choices and effort—a manipulation not possible to manage in the present experiment.

Were the three reinforcement schedules used in this experiment psychologically equivalent for pigeons? With the present design, for example, it was impossible to know whether a reduction of delay from 12 to 6 s was similar to an increase in reward probability from 0.33 to 0.66. We could only show *a posteriori* that they responded similarly in both cases. We fixed the delay decrement and probability increment values based on Wittek et al. (2021, Experiment 1), where it was found that well-trained pigeons gave approximately 20 pecks to an 8-s CS and that they kept constant pecking rates when the CS duration was extended to 24 s. On this basis, we could estimate the number of pecks to a 12-s CS to be around 30. The decrement and increment values were chosen to similarly improve the different options for the pigeons. With a delay decrement of 0.5 s per peck, the delay (to food or information) could be reduced by half with 12 pecks given in 6 s. With a probability increment of 0.015 per peck, the default reward probability (0.33) could be doubled with 22 pecks given in 12 s, that is, 12 pecks given in 6 s approximately. Thus, the three conditions were equivalent in terms of the effort requested to obtain a reward twice faster or twice more likely than in the absence of pecking. Despite equivalence between the three conditions relative to the pecking time available, pigeons might have been sensitive to the absolute number of pecks. They would not have favored the probability-increase option because they had to give approximately twice as many pecks as in the other two options.

The absence of variation in the decrement and increment values prevented us from drawing general conclusions about the psychological preference and pecking investment of pigeons between the three reinforcement schedules. We only observed that preference and effort do not necessarily work in concert and that profitability plays little role in the pigeons’ decisions *with the chosen values*. But our goal was to determine, for those chosen values, how the pigeons’ food restriction level influenced their behavior. These effects might have greater generality, even in the absence of systematic comparisons (e.g., Gabriel et al., 2021).

In conclusion, pigeons shied away from investing much effort to increase reward probability and thus did not peck more for this option than for delay reduction, despite the low initial probability of food in both options. But delay reduction was preferred over probability increase. Thus, pigeons preferred to know as soon as possible whether food was coming, although they did not invest more effort for it. These results may suggest (a) that the ecology of pigeons favor a preference for shorter delays to food and (b) that the presence of residual uncertainty for each option in our task induced similar effort irrespective of the amount of food received over a session. Similar paradoxes between preference and effort might happen in the wild and for many reasons—e.g., food in area A is preferred to food in area B, but more effort is produced in area B because of the presence of predators in area A. These analyses of choice patterns in pigeons reveal that psychological theories of choice have to be constrained by species-specific ecological conditions.

## Data availability statement

The raw data supporting the conclusions of this article will be made available by the authors, without undue reservation.

## Ethics statement

The animal study was approved by Landesamt für Natur, Umwelt, und Verbraucherschutz Nordrein Westfalen. The study was conducted in accordance with the local legislation and institutional requirements.

## Author contributions

NW: Software, Writing – review & editing. BS: Writing – review & editing. NO: Writing – review & editing. KW: Software, Writing – review & editing. NG: Writing – review & editing. FO: Writing – review & editing. OG: Data curation, Supervision, Validation, Writing – review & editing. PA: Conceptualization, Data curation, Formal analysis, Funding acquisition, Investigation, Methodology, Project administration, Resources, Supervision, Validation, Visualization, Writing – original draft, Writing – review & editing.
